# The Role of Brown Adipose Tissue Dysfunction in the Development of Cardiovascular Disease

**DOI:** 10.3389/fendo.2021.652246

**Published:** 2021-05-25

**Authors:** Hong-Jin Chen, Ting Meng, Ping-Jin Gao, Cheng-Chao Ruan

**Affiliations:** ^1^ Department of Cardiovascular Medicine, State Key Laboratory of Medical Genomics, Shanghai Key Laboratory of Hypertension, Department of Hypertension, Ruijin Hospital, Shanghai, China; ^2^ Shanghai Institute of Hypertension, Shanghai Jiao Tong University School of Medicine, Shanghai, China; ^3^ Shanghai Key Laboratory of Bioactive Small Molecules, Department of Physiology and Pathophysiology, School of Basic Medical Sciences, Fudan University, Shanghai, China

**Keywords:** brown adipose tissue, cardiovascular diseases, obesity, adipokines, adipocyte

## Abstract

Brown adipose tissue (BAT), consisted of brown adipocytes and stromal vascular fraction, which includes endothelial cells, lymphocytes, fibroblasts and stem cells, plays a vital role in regulating cardiovascular health and diseases. As a thermogenic organ, BAT can influence body through strengthening energy expenditure by promoting glucose and lipid metabolism. In addition, BAT is also an endocrine organ which is able to secret adipokines in an autocrine and/or paracrine fashion. BAT plays a protective role in cardiovascular system through attenuating cardiac remodeling and suppressing inflammatory response. In this review, we summarize the advances from the discovery of BAT to the present and provide an overview on the role of BAT dysfunction in cardiovascular diseases.

## Introduction

Since 2000, the prevalence of obesity has risen sharply around the world. According to the research, a total of 108 million children and 604 million adults had been obese by 2015. Obesity accounted for 2.4 million deaths globally, and more than two thirds of deaths were due to cardiovascular disease (CVD), including hypertension, coronary heart disease and stroke (1). In particular, the accumulation of visceral fat can greatly increase the risk of death from CVD (2). These patients experience systemic changes, especially the change of white adipose tissue (WAT). WAT is a vital organ in energy storage, which can excessively accumulate in obese patients (3). The increase of WAT is the result of both hyperplasia and hypertrophy of adipocytes (4). In addition to WAT, there are two types of adipose tissue: brown adipose tissue (BAT) and beige adipose tissue. BAT merely represents 1–2% of fat, consisting of brown adipocytes and stromal vascular fraction (SVF), but it is vital in maintaining homeostasis. BAT has a crucial capacity for non-shivering thermogenesis in mammals, which is essential for survival in cold environment and consumption of excessive energy. Recently, research has found that BAT is not only significant in maintaining cardiovascular stability, but also recognized as a novel target to deal with obesity and other metabolic diseases, which attracts more and more attention. In this article, we review the development of BAT in animal models and human, discussing its relevance to cardiovascular damage.

## Discovery And Location Of Bat

Early in 1551, BAT was first discovered in the scapula of a groundhog by Swiss naturalist Konrad Gessner, which was thought to be a gland associated with hibernation. It was not until twentieth century that BAT was considered as a mature tissue with certain component. Later, through necropsy studies, Heaton found BAT mainly located in (1) cervical/axillary (2), perirenal/adrenal, and (3) around blood vessels. In addition, BAT was also found in the scapula of infants, which was the major location in rodents (5). In infants, BAT accounts for about 2–5% of body weight, while in adults, it only accounts for 0.05–0.1%. The amount of BAT will decline with age, but it remains in specific areas of human body all the time. There are some researches showing that individuals exposed to cold will possess more BAT (6–8). However, lacking valid non-intervention means, the studies on BAT *in vivo* have been greatly limited for a long time. It was not until the application of positron emission tomography (PET) in combination with computer tomography (CT) that rekindled people’s interest in BAT research. In 2009, three papers published in NEJM confirmed the presence of functional BAT in healthy humans, and the location was consistent with previous autopsy findings (9–11). Virtanen’s research found a 15-fold increase in glucose intake in the cervical and supraclavicular regions of five participants exposed to cold (9). Cypess et al. reported that women possessed more BAT than men (10). According to the research, except for classic BAT, some BAT is mixed with WAT rather than presents alone (12). The aforementioned beige adipose tissue is also contained in WAT. Beige adipocytes intersperse within WAT, which can transforming into brown-like adipocytes under the certain stimulation such as cold exposure and beta adrenaline (13, 14). This process is named browning.

## The Origin Of Bat

There was a research that researchers removed 40% BAT from young male rats surgically showing that the total mass, the oxidative and thermogenic capacity of BAT in experimental group was identical to control group after 9 days (15). There may be two possibilities. One is the functional compensation of remaining BAT. The other reason may be the differentiation of preadipocytes. A variety of researches have focused on the origin of BAT over the past decade. It is well-accepted that brown adipocytes share the same lineage with skeletal muscle cells. Pax3 and Pax7-expressing cells are confirmed to be the progenitor cells of skeletal muscle (16). And the function of Pax3 can be replaced by Pax7 mostly (17). Later in 2010, Pax7+ cells at embryonic day 9.5 (E9.5) can give rise to brown adipose tissue. While after E11.5, the Pax7 marked cells reduced drastically in BAT and then disappeared in E12.5 (18). As the embryo develops, Pax7-expressing cells become restricted to muscle-specific fate. Myf5 is also proved to be expressed in skeletal myogenic precursors previously, which plays an important role in myogenic determination (19). Then Myf5-expressing cells were confirmed by Seale et al. that they could give rise to brown adipocytes in the development of classic BAT, beige adipocytes not included, while Myf5 mRNA was not found in mature BAT (20). Brown adipocytes arise from multiple lineages. Classic brown adipocytes are mentioned above that they are broadly believed from Pax7/Myf5 progenitor cells, while these genes just expressing transiently in the development of BAT. Activated beige adipocytes are similar to brown adipocytes. They are distributed in many areas, including WAT, PVAT and so on. Thereinto, SM22α is reported to take part in the development of perivascular adipocytes transiently (21). Recent research shows that the adipocytes in thoracic aorta perivascular adipose tissue (T-PVAT) have different cell lineages. In this research, T-PVAT is divided into anterior T-PVAT (A-T-PVAT) and lateral T-PVAT (L-T-PVAT), and the results suggest that A-T-PVAT adipocytes are derived from SM22α progenitors while L-T-PVAT adipocytes are originated from cells containing both SM22α and Myf5 (22). Different sources of adipose cells may indicate they will play different roles in maintaining body homeostasis.

## Functions Of Bat

### Metabolic Function of BAT

Since its discovery, BAT, as a thermogenesis organ, has been linked to heat production, which is regulated by the sympathetic nervous system. Thermogenesis is a manifestation of metabolic process containing shivering thermogenesis and non-shivering thermogenesis. Thereinto, shivering thermogenesis is the main contributor to heat generation under the circumstance of extreme cold. It is the result of involuntary contraction of skeletal muscles (23), and this process requires a lot of energy, causing discomfort and fatigue. Non-shivering thermogenesis under cold stimulation is to activate sympathetic nervous system to promote BAT heat production (24). Inhibiting the thermogenic function of BAT by using nicotinic acid led to increased muscle contraction against cold temperature, which demonstrated that BAT plays an important role in maintaining a normal body temperature in cold (25). Brown adipocytes with multilocular lipid droplets, are rich in mitochondria, and can significantly express uncoupling protein 1 (UCP1), PGC1α, PR domain-containing protein 16 (PRDM16) (26, 27), β3-adrenoceptor and other genes related to thermogenesis. Activated BAT expresses β3 adrenoreceptors which mediate the sympathetic drive to mobilize and upregulate UCP1 to promote a large amount of energy loss in the form of heat energy (28). Thus BAT plays a vital role in body energy expenditure through increasing glucose metabolism and lipid metabolism, which may be a valuable therapeutic approach to metabolism-related diseases, such as obesity. According to the research, nearly 40 g totally activated BAT in man could correspond to as much as 20% of body energy expenditure over a year, which is equivalent to 20 kg of body weight (29). Hence BAT is an important regulator not only in energy metabolism, but in the lipid and glucose metabolism and these two aspects are interrelated. Free fatty acids are the main source of oxidation in BAT to produce heat which is from the lipolysis of the triglyceride (TG) in lipid droplets in adipocytes. With the oxidation of fatty acids, reduced TG needs to be restored through the uptake of glucose and albumin-bound free fatty acid in the plasma (30) in order to provide the source of mitochondrial oxidation. Thus activated BAT has its place in the clearance of glucose and TG in the plasma (31, 32). According to the research, the utilize of glucose in BAT accounts for nearly 1% of the total body glucose use, and that is about 5g of glucose in a healthy individual (33). Disordered glucose and lipid metabolism including decreased high-density lipoprotein (HDL-C), increased triglyceride-rich lipoprotein and insulin resistance are important risk factors for CVD (34). As mentioned above, BAT accounts for 0.05–0.1% of body weight in adults. Thus a healthy adult possesses nearly 40 g BAT, which plays a vital role in balancing body energy, lipid and glucose metabolism.

### Secretion Function of BAT

Apart from thermogenesis, BAT has gradually attracted more attention as a secretory organ. Before that, a number of WAT-secreted molecules, which are called adipokines, have been identified in recent years, including inflammatory cytokines, leptin and so on. However, these adipokines are rarely expressed in BAT, which led the researchers to think that BAT has limited function of secretion (35). Recent years, the application of proteomics analysis in BAT researches provides researchers with an effective method to discover new cytokines. Ail et al. found fibroblast growth factor 21 (FGF21), interleukin-6 (IL-6), neuregulin-4 (NRG4) and vascular endothelial growth factor A (VEGFA), expressed in BAT will involve in thermogenesis, angiogenesis and the browning of WAT in an autocrine and/or paracrine fashion (36–40). Brown adipokines, they mainly act on different tissues or target organs to protect or regulate the cardiovascular system. For example, FGF21 is shown to have an important protective effect on the heart (41); IL-6, whose concentration is usually considered as indication of inflammatory response (42), has a positive function on regulating the glucose metabolism of BAT working together with FGF21 (43, 44) and the function of anti-inflammation (45). In addition, IL-6 can perform completely different function depending on cell type and context; bone-morphogenetic protein 8b (BMP8b) takes part in the neurovascular remolding (46); NRG4 has a negative relation with acute coronary syndrome (ACS) (47); C-X-C motif chemokine ligand-14 (CXCL14) and growth differentiation factor 15 (GDF15) participate in anti-inflammation process (48, 49). Furthermore, there are several bioactive lipid termed lipokines from both WAT and BAT, which also play an important role in the regulation of cardiovascular health. Recently, a novel lipokine derived from BAT, 12,13-dihydroxy-9Z-octadecenoic acid (12,13-diHOME), has been confirmed to play a positive role in cardiac function (50). However, further research is needed on the discovery and characteristics of the new cytokines derived from brown adipocytes. The important factors, which have been found discovered so far, are listed in **Table 1**.

**Table 1:** Major factors expressed by adipose tissue and their important and putative functions.

Factors	Origin	Regulation	Putative functions
	WAT		
	BAT		
Leptin	+ +	Increased AT mass↑ Fasting↓ β-adrenergic activator↓ Obesity↑	**In physiological conditions:** Decrease atherosclerosis Decrease insulin secretion Increase energy expenditure In pathological conditions: Increase insulin resistance Hyperglycemia Decrease thyroid hormone Increase neointima formation
FGF21 ⭐	− +	β-adrenergic activator↑ Cold exposure↑ Obesity↑	**In physiological conditions:** Increase insulin sensitivity Increase browning of AT In pathological conditions: Decrease cardiac hypertrophy Decrease cardiac fibrosis
IL-6	+ +	Psychological stress↑ β-adrenergic activator↑ BAT transplantation↑	**In physiological conditions:** Decrease inflammation Increase energy expenditure Increase lipolysis Increase insulin sensitivity In pathological conditions: Increase inflammation Increase insulin resistance
NRG4	− +	Obesity↓ Increased carotid intima-media thickness↓ Increased atherosclerotic plague↓	**In physiological conditions:** Improve insulin resistance Decrease lipogenesis Increase cardiomyocyte proliferation Decrease inflammation Decrease apoptosis of endothelial cell Decrease apoptosis and necrosis In pathological conditions: Increase insulin resistance Increase inflammation
VEGFA	+ +	Cold exposure↑	**In physiological conditions:** Increase angiogenesis Improve metabolic dysfunction Increase browning of AT Increase energy expenditure Decrease inflammation In pathological conditions: Increase inflammation
CXCL14	− +	Cold exposure↑ Norepinephrine↑ cAMP↑ Obesity↓	**In physiological conditions:** Promote the recruitment of M2-type macrophage Increase browning of AT In pathological conditions: Decrease glucose tolerance Increase inflammation
GDF15⭐	+ +	Cold exposure↑ CVD↑ Cancer↑	**In physiological conditions:** Increase lipolysis Improve insulin resistance In pathological conditions: Decrease inflammation
PGC-1α	− +	Cold exposure↑ Norepinephrine↑ Aging↓	**In physiological conditions:** Improve vascular senescence Decrease inflammation Decrease oxidative stress Regulate mitochondrial biogenesis In pathological conditions: Increase inflammation Increase oxidative stress
PRDM16	+ +	Aging↓	**In physiological conditions:** Increase browning of AT Regulate thermogenesis Suppress fibrogenesis Increase lipolysis Increase ketogenesis In pathological conditions: Increase browning of AT
Adiponectin	+ +	obesity↓ β-adrenergic activator↑ Calorie restriction↑ Increased bone marrow adipose tissue mass↑ Coronary artery diseases↓	**In physiological conditions:** Decrease inflammation Increase insulin sensitivity Decrease atherosclerosis In pathological conditions: Increase β-cell apoptosis
12,13-diHOME	− +	BAT transplantation↑ Heart diseases↓	**In physiological conditions:** Regulate calcium cycling Increase mitochondrial respiration In pathological conditions: Decrease cardiac protection

− represents this factor is not expressed or rare expressed in the tissue; + represents this factor is expressed in physical condition or certain conditions. Most factors listed above play a protective role in physiological conditions, and their expression decrease in pathological conditions which leads to bad results as showed in “in pathological conditions” column. “*” means the expression of these factors will increase early in pathological conditions and then exert protective effects.

## Bat and Cardiovascular Diseases

**Figure 1:**
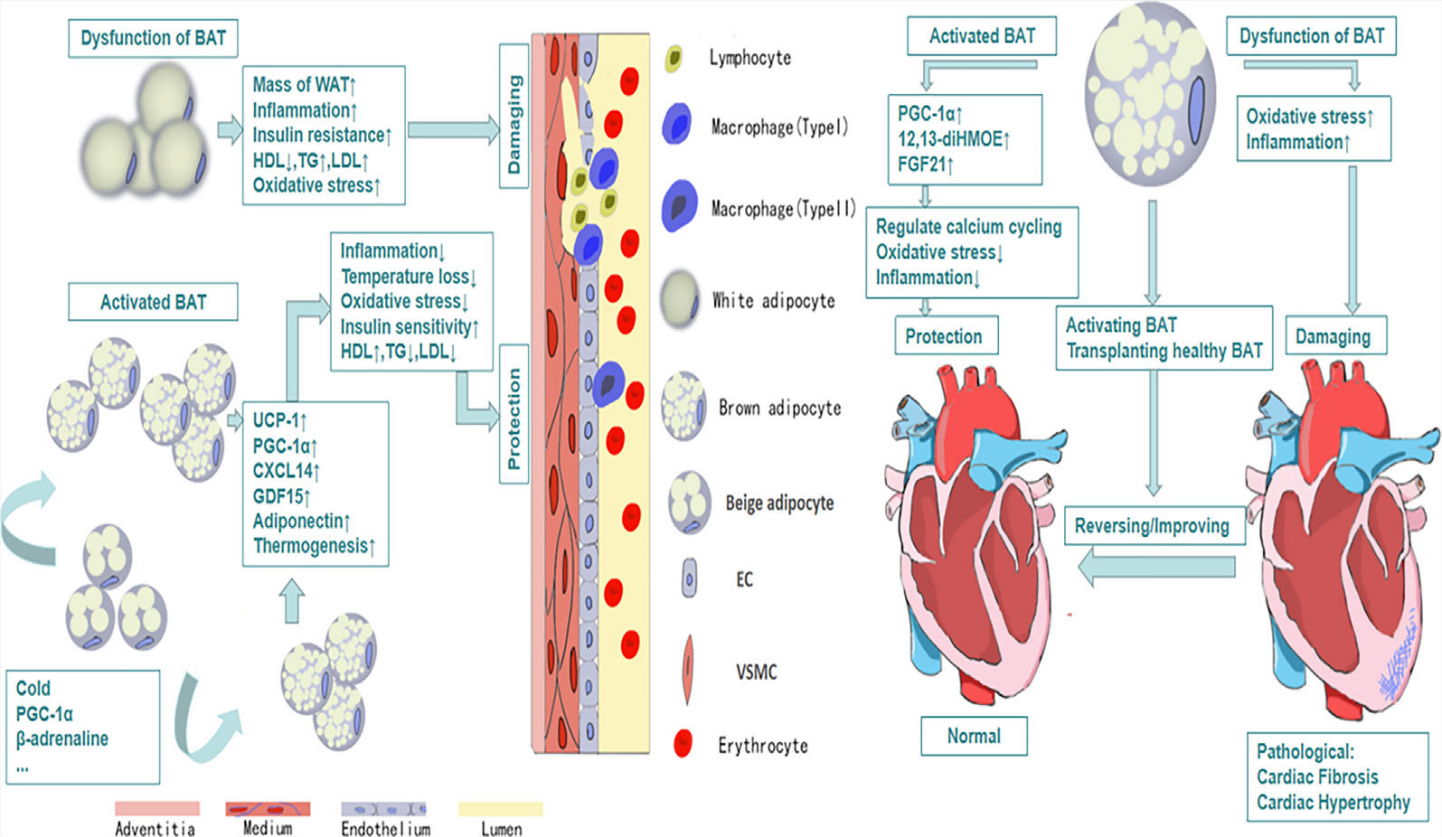
The protective efforts of BAT on vessels and heart. Activated BAT, including classic BAT and the BAT derived from the browning of beige adipocytes under the condition of cold exposure, beta-adrenaline stimulation and so on, plays a protective role through its metabolic function and secretion function, which leads to decreased inflammation, decreased oxidative stress response and increased HDL, increased uptake of glucose and so on. And all of these contribute to cardiovascular health and vice versa.

The relationship between obesity and cardiovascular diseases has received much attention since last century (51). Multiple epidemiological investigations indicate that obesity is the major determinant of cardiovascular diseases especially in adolescents (52). More than 75% high blood pressure is caused by obesity directly (53). A large number of studies have reported that there are structural and functional heart abnormalities in obese subjects, such as left atrium enlargement and left ventricular hypertrophy (54–56). There are also some researches showing that the activity of BAT will decline in obesity (57), which may reveal that BAT plays a positive role in health maintaining (**Figure 1**). Active BAT may promote cardiac metabolic health through the combustion of triglycerides and glucose derived free fatty acids, thus preventing adipose tissue dysfunction, obesity and insulin resistance (58). As early as the twentieth century, Cittadini et al. developed obese mice based on the ablation of BAT by transgenic technology to study the relationship between BAT and CVD. In their research, they found these knockout mice had decreased energy-expenditure, and hyperphagia leading to obesity, composed with decreased body temperature and metabolic rate. Their follow-up research showed that in addition to the development of obesity and insulin resistance, the ablation of BAT led to the elevation of blood pressure, left ventricular hypertrophy with an eccentric remodeling pattern and increased interstitial tissue (59). In clinical research, Richard and his group’s data obtained from the follow-up investigation of 443 patients indicated that the activity of BAT had a negative relationship with vascular inflammation and CVD (60). And the beneficial effects of BAT on improving blood glucose, TG and HDL play an significant role in promoting cadiometabolic health (61). The next question for the researchers is how BAT is involved into the occurrence and development of CVD.

### BAT and Vascular Injury

Vascular injury refers to the structural damage or dysfunction of blood vessel caused by mechanical or chemical stimulation *in vitro* or *in vivo*. And atherosclerosis and aorta aneurysm, considered as the two most common and dangerous human diseases, are given serious attention.

Atherosclerosis is associated with a chronic inflammation reaction, including the accumulation of lipid, infiltration of inflammatory cells, proliferation, migration of smooth muscle cells, oxidative stress and neovascularization. Obesity is a recognized risk factor for atherosclerosis. Adipose tissue, including WAT,BAT and thoracic and abdominal PVAT, play an important role in the occurrence and development of atherosclerosis. It is traditionally believed that the increased low density lipoprotein (LDL) in the blood of obese people, which is deposited on the wall of blood vessels, then swallowed by macrophage and finally forms the foam cells, is the key step in the formation and development of atherosclerosis (62). However, recent researches show that the formation of atherosclerotic plaque is initially due to endothelial dysfunction, which is associated with infiltration of inflammatory cells, caused by the pro-inflammatory factor secreted by adipose tissue (63, 64). In animal studies, researchers use beta-adrenaline to induce browning of adipose tissue. Increased BAT can slow the development of hypercholesterolaemia and atherosclerosis in hyperlipidemia mice (65). Mitochondria are abundant in BAT, which can synthesize and release peroxisome proliferator-activated receptor gamma coactivator-1α (PGC-1α) to assist carbon monoxide to complete vasodilation. The decreased BAT results in the insufficient synthesis of PGC-1α and then impairs vasodilation seriously (66). In addition, PGC-1α plays a vital role in the biosynthesis and function of mitochondria, and the dysfunction of mitochondria will cause a series of problems including telomere dysfunction, DNA damage and oxidative stress (67). There are also evidence showing that PGC-1α can regulate vascular senescence negatively (68). In the study of atherosclerosis, there are researches showing that the BAT-derived exosome can inhibit the increase of miR-324-5p, which is the specific biomarker of the development of atherosclerosis (69). There are also many other BAT-derived cytokines involved in vascular health. Adiponectin, for example, which is produced by adipose tissue and enters into the circulation, was suggested to fight against atherosclerosis through suppressing endothelial inflammation and VSMC proliferation. Besides, it can restrain the transformation of macrophage to foam cells (70). Apart from BAT, the adipose tissue around vessels, which is called perivascular adipose tissue (PVAT), also plays a vital role in protecting vessels. PVAT, especially thoracic PVAT, similar to BAT, is a thermogenesis organ, which is crucial for maintenance of intravascular temperature. The activation of PVAT can attenuate the development of atherosclerosis, through preventing intravascular temperature loss which can directly maintenance the function of endothelial. Furthermore, thermogenic activation of PVAT can enhance the clearance of total lipid. While this protection will disappear when PVAT is removed (21). In addition, there is an underestimated type of adipose tissue, called epicardial adipose tissue (EAT), increasing of whose mass is considered as a risk factor for the development of coronary artery diseases (71). EAT is considered as a type of beige adipose tissue with overexpression of UCP-1, the marker of brown adipose tissue, relative to WAT (72). Akin to PVAT, EAT possesses significant thermogenic capacity, sharing a negative association with temperature, and plays an important role in protecting cardiovascular health. α2A-adrenergic receptor (ADRA2A) is an inhibitory α-adrenergic receptor, which is at a lower level compared to WAT and it may contribute to the higher signaling *via* β-adrenergic receptors in EAT. Besides, through adding conditioned media which was collected from EAT treated with isoproterenol to primary human cardiac endothelial cell and then culturing it for 24 h, significant down-regulation of the expression of adhesion markers such as Icam1 and Vcam1 in endothelial cells were detected compared to control group (73). Thus abnormal EAT affected the function of endothelial cells seriously which is considered as the initial pathological process in the development of atherosclerosis. What’s more, research reported that activated EAT was associated with decreased circulating TG levels and increased HDL-C levels which had protective effect on atherosclerosis (72). In conclusion, in obesity, increased fatty acid release by WAT and decreased lipid combustion by BAT and thoracic PVAT lead to hyperlipidemia, which contributes to atherosclerosis development. Besides, obese WAT and abdominal PVAT release pro-inflaammatory factors that further promote atherosclerosis (74).

Aortic aneurysm (AA), defined as a pathological and progressive dilation of a segment of a blood vessel, is a common and dangerous vascular disease, especially abdominal aortic aneurysm (AAA). There is still lacking effective means of medical treatments. For the pathogenesis of AA, the mainstream view is that decreased smooth muscle cells, the degradation of cell matrix and infiltration of inflammatory cells in the blood vessel result in the thinning of adventitia and media jointly (75). The high risk of cardiovascular-related death in obesity is due to the AA partly (76). In the research, researchers found obesity could increase the morbidity of AngII-induced AA and exacerbate perivascular infiltration of macrophage and expression of MCP-1, IL-6, chemotactic factors and so on. In addition, they found there were more brown adipocytes around thoracic aorta, while more white adipocytes around abdominal aorta. Thus they finally draw the conclusion that in obesity, the high incidence of AA, especially AAA, is critically due to the decreased BAT which aggravates vascular inflammation rather than changes cholesterol concentration, distribution of lipoprotein and insulin resistance in blood (77). Dowal and his group confirm that BAT has ability to repress the inflammatory action of macrophage (78). Further research shows that BAT can inhibit inflammation to protect blood vessels through following two aspects at least. On the one hand, BAT-derived CXCL14 can recruit M2 macrophage possessing anti-inflammation ability (48). On the other hand, BAT can secrete GDF15 which acts on M1 macrophage to inhibit its pro-inflammation response (49).

In summary, the capacity of BAT in vascular protection is now widely recognized to be multifaceted from its original thermogenic function to its powerful endocrine function and metabolic function. And its anti-inflammation function and the ability to improve lipid, glucose metabolism can prevent of reverse vascular diseases directly or indirectly.

### BAT and Heart Diseases

Heart failure, as the terminal state of kinds of heart diseases, with high mortality and bad prognosis, is a clinical syndrome caused by the structural or functional abnormalities of the heart. It is a progressive disease, from initial myocardial damage to the damage of ventricular filling and cardiac ejection, and then to ventricular remolding finally. Recent research shows that adipose tissue-derived cytokines take part in the regulation of heart diseases (79). In animal study, removing BAT in the body will aggravate cardiac remolding, which appears BAT may play a role in cardiac protection in some way. FGF21, as one of the first proven cytokines from BAT, has a protective effect on the heart. And in the follow-up study, activation of adenosine 2A (A2A) receptor in BAT can mediate BAT secrete FGF21, which can attenuate hypertensive cardiac remolding (80). In addition, mice with UCP-1 knockout had severe myocardial injuries, fibrosis and ventricular remolding when they were given heart injury in experiments, and their survival rate was significant reducing. However, transplanting BAT from healthy mice could reverse heart damage and improve survival (81). As is mentioned above, PGC-1α plays an important role in vascular protection. It is essential for heart protection as well. The dysfunction of BAT results in the decrease of PGC-1α synthesis. In some patients with cardiac hypertrophy caused by hypertension or heart failure, it was found that reduced PGC-1α would lead to the decrease of the oxidation of fatty acid and the suppression in mitochondrial oxidative phosphorylation (82). While the exact mechanisms have not been elucidated. Some researches show that BAT can inhibit NF-κB through SIRT1-PGC1α-PPARγ pathway to suppress inflammation (83). What’s more, Pinckard et al. found that the transplantation of BAT could reverse cardiac dysfunction and cardiac remodeling caused by high-fat diet through increase the expression of 12,13-diHOME. Further research showed that 12,13-diHOME together with NOS1 could enhance cardiac function by regulating the calcium cycling (50). In addition, the dysfunction of adipose tissue has been discussed on a variety of cardiac disorders in many researches (84).

### The Negative Effect of BAT

There are a number of studies showing that BAT plays a positive role in cardiovascular damage. While some researches show that activated BAT and increased browning of WAT may exacerbate atherosclerosis (85, 86). Activated BAT and increased beige adipose tissue can elevate lipolysis and thermogenesis, then increase bio-synthesis of cholesterol and mobilization of low density lipoprotein (LDL) and very low density lipoprotein (VLDL), which promotes the growth of atherosclerotic plague. In their further study, they found genetic deletion of UCP1 in ApoE(−/−) mice, which is the specific marker of BAT, could prevent the occurrence of the above phenomenon.

## Conclusion

It is widely accepted that obesity can induce and accelerate the progression of CVD. Traditionally, the accumulation of WAT in obese people was considered as the major factor that increases the morbidity of CVD, while the role of BAT in obesity has been paid much more attention recently. In the past, due to the lack of accurate detection methods, the clinic value of BAT has been greatly underestimated. With the development of technology, such as the application of PET/CT, BAT is strongly associated with health. After kinds of studies, BAT is considered as not only a thermogenic organ, but also a endocrine organ. It has a powerful anti-inflammatory effect which plays an important role in cardiovascular protection. As a bridge between CVD and metabolic diseases, BAT will provide new ideas for the treatment. While recent researches show activated BAT is not as good as we thought before, which can accelerate the formation of atherosclerosis. So BAT might be a double-edged sword in the development of CVD and the exact mechanism of BAT has not been understood yet. Thus developing its positive function fully and reducing its possible negative effects are the direction we should strive for in the future, and it needs further elaboration of its possible mechanism in the development of CVD.

## Author Contributions

C-CR and H-JC designed the study and wrote the manuscript. TM and P-JG performed critical revision of the manuscript. All authors contributed to the article and approved the submitted version.

## Funding

This work was supported by the National Natural Science Foundation of China (81922004, 81770495, 91739303, 81870180 and 81700433) and the Natural Science Foundation of Shanghai, China (19JC1414600).

## Conflict of Interest

The authors declare that the research was conducted in the absence of any commercial or financial relationships that could be construed as a potential conflict of interest.
